# Positive and negative affect influence learning during exposure therapy: Secondary analysis from a randomized controlled trial

**DOI:** 10.1016/j.janxdis.2026.103205

**Published:** 2026-06-23

**Authors:** Benjamin M. Rosenberg, Michael Treanor, Tomislav D. Zbozinek, Christina F. Sandman, Amy Sewart, Michelle G. Craske

**Affiliations:** a Neuroscience Department, Pomona College, Claremont, CA 91711, USA; b Department of Psychiatry and Biobehavioral Sciences, University of California, Los Angeles (UCLA), Los Angeles, CA, USA; c Department of Psychology, University of California, Los Angeles (UCLA), Los Angeles, CA, USA

**Keywords:** Exposure therapy, Anxiety, Negative affect, Positive affect, Anhedonia

## Abstract

**Objective:**

The Inhibitory Retrieval model of exposure therapy emphasizes expectancy change as a core mechanism of treatment. Evidence suggests important roles for both positive and negative affect during exposure therapy. The current study assesses the extent to which positive and negative affect moderate expectancy change during exposure therapy.

**Method:**

N =77 adults with either social anxiety disorder or panic disorder completed a nine-session exposure therapy protocol: n =41 were randomized to exposure therapy based on principles of extinction learning (Inhibitory Retrieval condition), during which they rated expectancy of an aversive outcome before and after each exposure; and n =36 participants were randomized to exposure focused on fear reduction (Habituation condition), during which they rated subjective experiences of fear before and after each exposure. The Positive and Negative Affect Schedule (past month) was collected at baseline, mid-treatment and post-treatment.

**Results:**

In the Inhibitory Retrieval condition, positive and negative affect each moderated the relationship between pre- and post-expectancy. Specifically, individuals with low positive affect relative to their own mean (*p* =.029) or high negative affect relative to other individuals (*p* <.001) reduced expectancies less when pre- expectancy was high. In the Habituation condition, neither positive nor negative affect moderated the relationship between pre- and post-exposure fear.

**Conclusions:**

Affect moderates expectancy change during exposure therapy modeled using Inhibitory Retrieval, such that higher negative affect and lower positive affect are associated with less expectancy change. Considering the historical emphasis on reducing negative affect, models of exposure therapy may additionally incorporate strategies to increase positive affect to maximize expectancy change.

## 1. Introduction

Exposure therapy is considered a gold-standard treatment for anxiety and related disorders (Abramowitz et al., 2019; McLean et al., 2022; Rauch & Ruzek, 2012; Reid et al., 2021). However, 50% or fewer individuals do not achieve clinically significant gains (Cuijpers et al., 2024), underscoring the need for additional research regarding moderators of therapeutic change. This study evaluates individual differences in positive affect (PA) and negative affect (NA) as potential moderators of expectancy change (a key purported mechanism of exposure therapy outcome) during exposure therapy for social anxiety disorder and panic disorder.

Exposure therapy involves repeated confrontation with feared stimuli (objects, situations, interoceptive cues, or memories) in the absence of the feared outcome. Procedurally, this is analogous to Pavlovian fear extinction, in which a conditional stimulus^4^ (CS) that was previously paired with an aversive outcome (unconditional stimulus, US) is repeatedly presented without the US. Extinction learning is driven by prediction error – or, a mismatch between high US expectancy and low actual rate of US occurrence (Rescorla & Wagner, 1972). Whereas earlier models emphasized unlearning of the excitatory CS-US association (Rescorla & Wagner, 1972), the inhibitory retrieval model of extinction posits that the original CS-US association acquired during fear conditioning is not erased, but rather is left intact as new, secondary learning about the CS-US association develops – specifically, that the CS no longer predicts the US in a given context (Bouton, 1993). Consequently, the CS possesses two meanings following extinction: the original excitatory meaning (CS-US) and a new inhibitory meaning (CS-noUS), with the latter gated by the context in which it was established.

Prediction error is maximized through expectancy violation, wherein anxious clients learn that feared stimuli (CSs) do not reliably predict feared outcomes (USs), thereby strengthening CS-noUS associations that compete with original CS-US associations (Craske et al., 2022). For example, an individual with social anxiety may expect that they will experience judgment or rejection in a given context (e.g., giving a presentation). Expectancy violation (e.g., experiencing no judgment or rejection) is thought to prompt the updating of predictions, or expectancy change, such that clients ultimately reduce their expectation of a CS-US pairing and extinguish their fear in that context (e.g., they predict a lower likelihood of judgment or rejection during the next presentation). Evidence increasingly supports that expectancy change is a core mechanism of exposure therapy that predicts long-term anxiety reduction. For example, in a study of over 8000 exposures across 605 patients with a wide range of anxiety disorders, Pittig et al. (2023) found that expectancy change, but not expectancy violation, predicted better outcomes during treatment. Furthermore, computational modeling of exposures for public speaking anxiety demonstrated that faster decreases in expectancy, over and above fear reduction, predict lower anxiety (Barnes-Horowitz et al., 2025). Understanding the moderators of expectancy change, therefore, may enable further optimizations to exposure therapy (Benito et al., 2024).

NA, which refers to unpleasant or distressing mood states associated with negatively valenced emotions (e.g., nervousness, fear, guilt), is highly relevant to fear extinction and exposure therapy. For example, individuals with anxiety show attentional biases toward negatively-valenced (e.g., threatening) environmental cues (Bar-Haim et al., 2007) as part of a bidirectional relationship with anxiety severity (Van Bockstaele et al., 2014). In particular, high “dispositional” or “tonic” (i.e., trait) NA is considered especially relevant to daily functioning and models of clinical anxiety (Shackman et al., 2016). For example, trait NA is a correlate (Böttger et al., 2023) and predictor of anxiety disorder onset (Jeronimus et al., 2016; Williams et al., 2021; Zinbarg et al., 2016). Trait NA also shows a positive, reciprocal relationship with anxiety and depression symptoms during CBT, suggesting that reductions to NA can drive treatment effects (Barnes-Horowitz et al., 2023). Considering that anxiety disorders are associated with high NA, and given the theoretical relevance of high NA during exposure exercises, NA is a strong candidate as a moderator of core mechanisms of exposure therapy, including expectancy change.

PA refers to a pleasurable mood state associated with positively valenced emotions (e.g., excited, happy, active, alert). Low PA is associated with anxiety disorders (Kashdan, 2007; Prenoveau et al., 2010) and predicts their onset (Kendall et al., 2015). Moreover, higher PA predicts more reduction in anxiety and depression throughout CBT, suggesting that changes to PA can drive treatment effects (Barnes-Horowitz et al., 2023). With respect to exposure therapy in particular, more positive emotionality predicts better treatment response (Taylor et al., 2023), whereas individuals with stronger anhedonia symptoms (a form of low PA involving reduced interest, pleasure, or motivation to engage in activities) still respond to exposure therapy but often over a longer course of treatment (Mattoni et al., 2025). One potential explanation is that low PA is associated with impairments in extinction learning and retention (Zbozinek & Craske, 2017b), including evidence for trait PA (Zbozinek & Craske, 2017a) as well as experimentally-induced momentary PA (Geschwind et al., 2015; Zbozinek et al., 2015). Likewise, anhedonia is associated with neural functioning during fear extinction (Rosenberg et al., 2023; Young et al., 2021) in ways that suggest impairments in the brain systems responsible for threat processing and US expectancy updating. Therefore, PA has been theorized to moderate core mechanisms of exposure therapy (e.g., expectancy change), such that increasing PA may enhance the efficacy of exposures (Rosenberg et al., 2024).

The present study was a secondary analysis of data collected for a randomized controlled trial comparing Inhibitory Retrieval and Habituation models of exposure therapy [ClinicalTrials.gov ID: NCT04048824] (Treanor et al., 2025). Although both NA and PA have theoretical relevance to expectancy change, a core mechanism of extinction learning during exposure therapy, no known studies have directly tested NA versus PA as moderators of expectancy change. Therefore, the goal of this study was to examine if NA or PA moderate expectancy change among individuals with social anxiety disorder or panic disorder. Principal hypotheses focused on the Inhibitory Retrieval condition. We hypothesized that NA and PA would each moderate expectancy change during exposures, such that high NA or low PA would be associated with less expectancy change. To investigate the specificity of effects to expectancy change relative to fear change, we conducted secondary analyses of NA and PA as moderators of fear change within the Habituation condition.

## 2. Materials and methods

### 2.1. Trial design

This study was a secondary data analysis of a parallel, two-arm, randomized (1:1), open label clinical superiority trial comparing inhibitory and habituation models of exposure therapy among individuals with social anxiety disorder or panic disorder (for details, see Treanor et al., 2025). The Institutional Review Board at the University of California, Los Angeles approved this trial. All participants signed informed consent.

### 2.2. Participants

Participant recruitment and screening is summarized fully in Treanor et al. (2025). A brief study description was distributed via lab websites, social media, ClinicalTrials.gov, and campus and local area flyers. The inclusion criteria for participation were age 18–65, English-speaking, seeking treatment for social anxiety disorder or panic disorder, and either stabilized on psychotropic medications or medication-free. Exclusion criteria were serious medical conditions, pregnancy, history of suicidal ideation or self-harm in the past year, history of suicide attempts in the last 10 years, history of bipolar disorder, psychosis, intellectual disability or organic brain damage, alcohol or substance use disorder within last 6 months, and concurrent therapy focused on anxiety. Screened participants underwent the Structured Clinical Interview for Diagnosis (SCID-V; First, 2014) – a semi-structured diagnostic interview, conducted by reliability-certified interviewers with diagnostic consensus led by experienced clinicians (MT or MGC). For diagnosed disorders, clinical severity ratings were provided, using a scale from 0 to 8 to indicate level of symptom severity, distress and impairment (Di Nardo & Barlow, 1988). Participants were eligible for inclusion if they received a clinical severity rating of 4 or higher for social anxiety disorder or panic disorder. This cutoff has been shown to yield rates of social anxiety disorder that are similar to prevalence estimates of social anxiety disorder in large-scale samples (Sewart et al., 2019).

Participants were included in analyses for the present study only if they had exposure data for at least one session (i.e., completion of session two) (n = 84/89 participants). Some participants were dropped due to missing questionnaire data across all three timepoints (n = 3 Inhibitory Retrieval; n = 4 Habituation) or due to missing exposure data across all three timepoints (n = 3 Inhibitory Retrieval, n = 2 Habituation). The final sample included N = 77 participants randomized to either the Inhibitory Retrieval condition (n = 41) or the Habituation condition (n = 36). Participant characteristics are summarized in Table 1.

### 2.3. Interventions

Interventions are summarized fully in Treanor et al., (2025). Treatment entailed nine weekly individual, manualized therapy sessions. The first session lasted 90 min and involved psychoeducation regarding the exposure therapy approach used in this study. Sessions two through nine lasted 60 min and involved therapist-guided in-session exposures and assignment of homework exposure practices. Therapy was provided by highly trained doctoral students in the UCLA Department of Psychology, supervised by licensed clinical psychologists (MGC and MT). Sessions took place in the Anxiety and Depression Research Center or remotely via an encrypted video-conferencing platform due to the COVID−19 pandemic. Participants were encouraged not to seek other forms of treatment (psychological or psychiatric) and to maintain their current medication regimen (if applicable) until completion of the nine-week treatment period.

In the Inhibitory Retrieval condition, participants first defined a nexus of factors influencing the likelihood of CS-US relationships (e.g., contexts or cues that make the US more or less likely to occur; Craske et al., 2022). Among those treated for social anxiety, the US was typically operationalized according to behavioral markers of rejection or judgement (e.g., explicit statements or facial expressions, or other pre-defined behaviors). Among those treated for panic disorder, the US was typically operationalized according to physical markers (e.g., heart attack, fainting). Exposure was therapist-directed during each session; participants first rated their expectancy of a US (PreExp), followed by an exposure activity designed to violate expectancies (see Craske et al., 2022, for details of the inhibitory retrieval model of exposure), consolidation of what they learned during the exposure, and finally a rating of expectancy for the US if they were to hypothetically repeat this exposure (PostExp). Participants were instructed to complete homework exposures to test the same CS-US association between sessions, although exposures varied stimuli and contextual elements to maximize extinction generalization.

In the Habituation condition, participants first constructed a fear hierarchy in ascending order of subjective fear ratings. During therapist-directed exposure sessions, participants first rated fear (PreFear), followed by an exposure activity repeated the number of times necessary for fear to reduce by 50% or a maximum of 45 mins, and finally a rating of fear following the last exposure (PostFear). Participants were instructed to complete the exact same exposures as homework between sessions.

### 2.4. Randomization and masking

Participants were randomized 1:1 to Inhibitory Retrieval vs. Habituation via computer-generated allocation using simple randomization. Randomization of participant ID numbers was created at the start of the study by the principal investigators. Only evaluators were masked to condition allocation. Study coordinators enrolled and assigned participants according to the randomization table.

### 2.5. Measures

Prior to beginning treatment, participants completed a two-day baseline assessment including the SCID−5 (First, 2014), behavioral assessments (see Supplement for details), and self-report questionnaires. NA and PA were measured using 41 items from the Positive and Negative Affect Schedule (PANAS-X; Watson & Clark, 1994), with ratings anchored over the preceding month. The Negative Affect subscale (PANAS-N) comprised items from the “General Dimension Scales – Negative Affect” and “Basic Negative Emotion” scales for Fear and Sadness (17 items, see Supplement). The Positive Affect subscale (PANAS-P) comprised items from the “General Dimension Scales – Positive Affect,” “Basic Positive Emotion” scales for Joviality, Self-Assurance, and Attentiveness, and “Serenity” scale (24 items, see Supplement). The PANAS was repeated at mid-treatment (week 5, following the fifth treatment session) and at post-treatment (week 10) along with behavioral measures and diagnostic evaluation. A subset of participants had PANAS data for 39–40 of the 41 items (baseline n = 5, mid-treatment n = 2, post-treatment n = 1). To include all participant data, PANAS data were converted to mean per-item scores for each participant at each time-point. Confirmatory factor analysis using the baseline PANAS scores revealed moderate-to-strong standardized factor loadings supporting the PANAS-N (range =.33–.79) and PANAS-P (range =.33–.84) scales used in this study.

For every exposure (therapist-guided in-session and self-guided between-sessions), participants in the Inhibitory Retrieval condition rated either their expected likelihood of the US occurring (“How likely is it that what I am most worried about will occur?”) on a scale from 0 (“Not at All”) to 100 (“Certain”). In contrast, participants in the Habituation condition rated their subjective fear (“Please use this 0-to−100 scale to rate your fear levels”) on a scale from 0 (“Not at All Afraid/Anxious”) to 100 (“Very Much Afraid/Anxious”). These values served as expectancy (PreExp or PostExp) or fear (PreFear or PostFear) ratings in subsequent analyses.

### 2.6. Statistical methods

The relationships between affect and exposure therapy mechanisms were evaluated using linear multilevel modeling (MLM) in Stata 18.0. MLM includes participants with missing data and uses all available data, which is recommended for clinical trials (Hamer & Simpson, 2009). All models included random effects of the intercept and fixed slopes for each participant. As PANAS ratings were completed for the preceding month at baseline (before session 1), mid-treatment (before session 5), and post-treatment (after session 9), we used US expectancy and fear ratings from therapy sessions two,^5^ five, and nine. Considering that exposure therapy has been previously linked to decreases in NA and modest increases in PA (Hoffman et al., 2024), analyses first tested for a main effect of Time in predicting PANAS-N and PANAS-P.

#### 2.6.1. Covariates

As exposure therapy during the trial was initially conducted in-person but then switched to remote therapy (due to COVID−19), all analyses controlled for Delivery (in-person, remote). To account for potential differences among those with social anxiety versus panic disorder, analyses additionally controlled for Treated Condition. Finally, as the intensity of exposures is designed to change over time during exposure therapy (i.e., PreExp or PreFear frequently increases) due to the progressive nature of exposures throughout treatment, all subsequent analyses controlled for Time.

#### 2.6.2. Principal analyses

Principal analyses were conducted within the Inhibitory Retrieval condition and focused on expectancy change. For sessions in which participants completed multiple exposure activities, we included all exposure data and included number of within-session exposures in each session as a covariate. Since differing levels of PreExp are likely to determine the relative size of expectancy change when calculated as a simple difference score (i.e., PreExp – PostExp), models tested if the association between PreExp and PostExp was moderated by NA or PA (i.e., PreExp x PANAS interaction predicting PostExp).

It is recommended that longitudinal multilevel models consider the individual as a “context” with both constant and momentary sources of variation (Hoffman, 2007; Hoffman & Stawski, 2009). This is particularly relevant to the measurement of affect over time, which is thought to capture elements of both state and trait affect (Brose et al., 2013; Kendall et al., 2015). Considering that the PANAS was completed over three one-month intervals, we decomposed the PANAS scores into sub-scales by calculating each participant’s mean PANAS-N or PANAS-P during treatment (between-participant effects) as well as mean-centered PANAS-N or PANAS-P values at each time-point of treatment (within-participant effects).

#### 2.6.3. Sensitivity analyses

To further disambiguate significant effects from the principal analyses, sensitivity analyses evaluated all PANAS-N effects over and above PANAS-P effects, and vice versa. Effects that are significant in the Principal Analyses but not in the Sensitivity Analyses would therefore be interpreted with additional caution.

#### 2.6.4. Secondary analyses

Secondary analyses were conducted within the Habituation condition and focused on fear change. In this condition, a single exposure ended when fear reduced by 50% or 45 min elapsed – whichever occurred first. Therefore, for sessions in which participants completed multiple repetitions of an exposure to sufficiently reduce fear ratings, we coded these repetitions as a single exposure exercise.^6^ Since differing levels of PreFear are likely to determine the relative size of fear change when calculated as a simple difference score (i.e., PreFear – PostFear), we accounted for relative differences in PreFear by calculating a PANAS x PreFear interaction predicting PostFear.

### 2.7. Transparency and openness

Treanor et al., (2025) describes how we determined our sample size, all data exclusions, all manipulations, and all measures in the study. The design and analytic plan for the RCT were pre-registered (ClinicalTrials NCT04048824). The specific hypotheses and analyses conducted for the present study were not pre-registered. Requests for deidentified data and research materials (including treatment manuals) should be directed to the corresponding author. Analysis code and corresponding raw outputs are provided in the Supplement.

## 3. Results

### 3.1. Changes in NA and PA over time

At Baseline, NA had a mean per-item score of 3.061 (SD=.049) and PA had a mean per-item score of 2.078 (SD=.042). In a typical sample, the mean per-item scores for NA are 1.600 and for PA are 3.131 (Crawford & Henry, 2004). Thus, baseline scores are consistent with elevated NA (above established norms) and diminished PA (below established norms) in this sample. There was a significant main effect of Time predicting NA (f=.740, Z = −5.99, *p* <.001) and PA (f=.707, Z = 5.84, *p* <.001), such that NA decreased and PA increased throughout exposure therapy. The distributions of NA and PA for each timepoint are included in the Supplement (see Fig. S1).

### 3.2. Associations between NA and PA

NA and PA were significantly negatively correlated at baseline (r = −.324, *p* = .044), but not at mid-treatment (r = −.111, *p* = .531) or post-treatment (r = −.110, *p* = .578).

### 3.3. Inhibitory retrieval

#### 3.3.1. Associations with NA

There was a significant PANAS-N x PreExp interaction, such that higher PANAS-N increased the effect of PreExp on PostExp (f=.384, Z = 4.44, *p* <.001; Fig. 1). Specifically, when PreExp was greatest, individuals who reported high NA showed the greatest PostExp (i.e., the smallest amount of expectancy updating). Within-participant variance in PANAS-N also significantly moderated the association between PreExp and PostExp (f=.183, Z = 1.96, *p* = .050), as did between-participant variance in PANAS-N (f=.350, Z = 4.11, *p* <.001).

#### 3.3.2. Associations with PA

There was a significant PANAS-P x PreExp interaction, such that higher PANAS-P decreased the effect of PreExp on PostExp (f=.159, Z = −2.06, *p* = .039; Fig. 2). Specifically, when PreExp was greatest, individuals who reported low PA showed the greatest PostExp (i.e., the smallest amount of expectancy updating). Within-participant variance in PANAS-P also significantly moderated the association between PreExp and PostExp (f=.199, Z = −2.54, *p* = .011). In contrast, between-participant variance in PANAS-P did not significantly moderate the association between PreExp and PostExp (f=.091, Z = −1.20, *p* = .231).

#### 3.3.3. Sensitivity analyses

The PANAS-N x PreExp interaction remained significant over and above PANAS-P effects (f=.332, Z = 3.77, *p* < .001). The effect for within-participant PANAS-N was no longer significant over and above PANAS-P effects (f=.103, Z = 0.98, *p* = .327), whereas the effect for between-participant PANAS-N remained significant in models controlling for PANAS-P effects (f=.364, Z = 4.34, *p* < .001).

The PANAS-P x PreExp interaction was no longer significant over and above PANAS-N effects (f=.020, Z = −.33, *p* = .739). However, the effect for within-participant PANAS-P remained significant over and above PANAS-N effects (f=.163, Z = −2.19, *p* = .029), while the effect for between-participant PANAS-P remained non-significant in models controlling for PANAS-N effects (f=.049, Z = 0.67, *p* = .505).

### 3.4. Habituation condition

#### 3.4.1. Associations with NA

PANAS-N did not moderate the relationship between PreFear and PostFear (f=.060, Z = −.40, *p* = .687). Neither within-participant variance in PANAS-N (f=.021, Z = −.05, *p* = .958) nor between-participant variance in PANAS-N (f=.067, Z = −.46, *p* = .649) moderated the relationship between PreFear and PostFear.

#### 3.4.2. Associations with PA

PANAS-P did not moderate the relationship between PreFear and PostFear (f=.096, Z = −1.17, *p* = .243). Neither within-participant variance in PANAS-P (f=.111, Z = 0.72, *p* = .473) nor between-participant variance in PANAS-P (f=.145, Z = −1.41, *p* = .159) moderated the relationship between PreFear and PostFear.

## 4. Discussion

This study evaluated learning mechanisms central to the efficacy of exposure therapy and their association with positive and negative affect. We found that both negative affect (NA) and positive affect (PA) moderated changes in expectations from before to after exposures that the anxiety-provoking situation (e.g., social anxiety: initiating a one-on-one conversation; panic disorder: elevating heart rate) would result in the aversive outcome (e.g., social anxiety: social rejection; panic disorder: heart attack). This effect occurred in the version of exposure therapy targeting expectancy change (i.e., Inhibitory Retrieval condition). Specifically, for exposures in which there was the highest expectancy of the aversive outcome, participants experiencing high NA or low PA were less likely to update their expectations following non-occurrence of that outcome. Follow-up and sensitivity analyses demonstrated that these effects were most attributable to between-participant NA (i.e., relative to other participants) and within-participant PA (i.e., relative to one’s own mean). Effect sizes were relatively larger for significant NA effects (f=.183–.384) versus significant PA effects (f=.159–.199), consistent with the historical emphasis on NA in the literature. PA may nonetheless warrant further attention as a target in exposure therapy. In contrast to the expectancy change results, NA and PA were not associated with fear change in a version of exposure therapy that targets fear reduction (i.e., Habituation condition).

### 4.1. Both NA and PA moderate expectancy change

Participants experiencing higher NA compared with other participants exhibited less updating of expectancy when expectations of the aversive outcome were highest. The association between NA and expectancy change fits within the broader literature linking NA to biased attention toward the detection of threats and reduced processing of stimulus and contextual factors (i.e., the “better-safe-than-sorry” phenomenon) (Van den Bergh et al., 2021) as well as general assumptions of threat as a default state unless inhibited by a safety signal (i.e., “generalized unsafety theory of stress”) (Brosschot et al., 2016, 2017). During exposures in which expectations of the aversive outcome are highest, high trait NA may therefore amplify avoidant tendencies or bias attention toward affect-congruent interpretations of continued threat, thereby undercutting extinction learning and maintaining high expectations of the aversive outcome. This phenomenon may be particularly evident when the aversive outcome is ambiguous and more reliant on interpretation, such as perceived rejection among those with social anxiety (i.e., the majority of our sample). Results suggest that a similar phenomenon may not occur when aversive outcome predictions are lower. However, as exposures in the Inhibitory Retrieval condition were specifically designed test moderate-to-high predictions of aversive outcome occurrence (i.e., 50% or greater), the present RCT was not optimized to test this possibility.

Furthermore, participants experiencing lower PA compared with their own mean exhibited less updating of expectancy when expectations of the US were highest. The association between PA and expectancy change fits within the literature linking PA to desirable learning outcomes, such as more stable long-term fear extinction (Zbozinek et al., 2015; Zbozinek & Craske, 2017a, 2017b). Furthermore, the positive emotion “relief” commonly occurs when a predicted aversive outcome does not occur, especially when the outcome is perceived as highly likely (Leng et al., 2023). Although strong experiences of relief can reinforce avoidance behaviors (i.e., high “relief-pleasantness”) (De Kleine et al., 2023; Vervliet et al., 2017), similar experiences of relief may also reinforce approach behaviors when an expected aversive outcome does not occur, as is commonly the case in exposure therapy (Rosenberg et al., 2024). Additional research is needed to confirm whether low PA is a robust predictor of lower expectancy change and, if so, to clarify the precise mechanism through which low PA reduces expectancy change.

### 4.2. Implications for theories of expectancy change

In the present study, NA and PA moderated expectancy change when expectancies for the US were highest. One possible explanation for this phenomenon is cognitive immunization theory, which posits that some beliefs do not diminish (or may even become stronger) when presented with new evidence (Kube et al., 2017; Rief et al., 2015). Although cognitive immunization has not been studied among individuals with anxiety disorders, this framework has been used to explain why positive disconfirming information is frequently interpreted with caution among those with depression (Kube et al., 2017, 2020, 2022; Kube & Glombiewski, 2021; Kube & Rozenkrantz, 2021), which frequently co-occurs with anxiety disorders (Kalin, 2020) and is also associated with both high NA and low PA (Hill et al., 2023; Medeiros et al., 2020). Relatedly, maladaptive emotion regulation strategies, such as dampening appraisals (e.g., “This is too good to be true”) (Kiken & Shook, 2014; Raes et al., 2009), are associated with both increases in NA and decreases in PA (Burr et al., 2017; Dunn et al., 2018). Considering the results from the present study, it is possible that clients with high NA or low PA are particularly susceptible to cognitive immunization, especially when expectancies are highest. Indeed, although exposures are typically designed with the explicit goal of generating positive disconfirming evidence, it is common for individuals to believe that an aversive outcome (e.g., social rejection) occurred even if there is no overt marker of this occurrence. This may be particularly common among individuals with social anxiety disorder who tend to show a negative interpretation bias in ambiguous contexts (Chen et al., 2020). For example, if a client engages in a public speaking exposure, it is possible the audience judged the client negatively even if no overt signs of judgment (e.g., explicitly negative statements, audience leaving in the middle) occurred. The client may therefore continue believing they were judged negatively (e.g., “They were a polite audience, but they still thought I was awkward”). Future research may test if these interpretations are more likely to occur when clients experience high NA or decreases in PA, thereby impeding inhibitory learning during exposure therapy.

### 4.3. Clinical implications & future directions

The findings from the present study have several clinical implications that warrant further investigation. Recent approaches to the treatment incorporate strategies to increase PA (e.g., Craske et al., 2019; for a review see Sandman & Craske, 2022). It may therefore be beneficial to incorporate these strategies prior to exposure therapy (i.e., adjunctive therapy) or during individual exposures (Rosenberg et al., 2024). Although the PANAS scale used in this study was not specifically designed to isolate state versus trait affect, we found that lower PA within- but not between-participants was associated with smaller expectancy change (specifically during exposures in which there was a high expectancy of an aversive outcome). One interpretation of the within-participant effect is that this measure may capture in-the-moment or state PA. If so, this might suggest that temporary positive mood inductions (e.g., watching a brief amusing video, pleasant mental imagery, or another enjoyable activity; Zbozinek et al., 2015) at the beginning of an exposure session could enhance learning and reduce cognitive immunization. Furthermore, if individuals experiencing low PA are more susceptible to cognitive immunization effects, therapists may provide psychoeducation about dampening appraisals and guide clients to mindfully disengage from these thoughts (e.g., to refocus attention on rewarding elements from the prior exposure or to gather more evidence via repeated exposures). For example, during the post-exposure discussion, in addition to asking about whether the aversive outcome occurred (Craske, et al., 2022), therapists could enhance experiential processing by asking clients about what was rewarding, enjoyable, or went well during the exposure (Sandman & Craske, 2024). These strategies may be particularly helpful when a client reports lower than their usual level of PA on weekly symptom questionnaires, or as PA continues increasing throughout the course of exposure therapy. These implications are tentative and require further study, especially considering that all PANAS ratings were provided based on the prior month (potentially limiting detection of state effects) and were measured during a course of psychotherapy (which is associated with decreases in negative affect and increases in positive affect). Likewise, clinical translation may especially benefit from a more specific analysis of individual positive emotions (e.g., Calm versus Proud) or PA sub-domains (e.g., Self-Assurance versus Attentiveness), as different interventions may engage distinct affective targets.

In contrast, considering that higher NA between- but not within-participants was associated with smaller expectancy change (specifically during exposures in which there was a high expectancy of an aversive outcome), this might suggest that momentary reductions in NA during exposure would not similarly enhance expectancy change. Indeed, strategies for reducing NA in-the-moment (e.g., safety behaviors) interfere with fear extinction (Lovibond et al., 2000) and are generally discouraged during exposure therapy, when possible (Blakey & Abramowitz, 2016). Instead of attempting to directly alter NA during an exposure, clients with high NA (compared with other clients) may instead benefit from focusing on exposures with moderate aversive outcome expectancies (e.g., 50%) during early phases of treatment. These exposures may elicit more openness to disconfirming information and therefore yield the largest changes in expectancy (in line with evidence among depressed individuals, see Kube et al., 2022). As NA reduces during the course of exposure therapy (Hoffman et al., 2024), more extreme exposures (i.e., with higher aversive outcome expectancies) may yield greater expectancy change during later stages of treatment.

Further research is needed to directly test these suggestions. Likewise, as the majority of studies have focused on strategies to reduce NA, additional work is needed to investigate positive emotion regulation and its relation to expectancy change during exposure therapy for anxiety disorders. Finally, future research into cognitive immunization effects among anxious or depressed samples should separate out the relative contributions of NA and PA to clarify the mechanisms underlying these associations.

### 4.4. Strengths & limitations

The present study has several strengths. First, by evaluating effects in both Inhibitory Retrieval and Habituation conditions, the study explores the effects of NA and PA that may be specific to expectancy change compared with fear change as mechanisms of exposure therapy. Second, the evaluation of NA over and above PA (and vice versa) enhances the interpretation that both PA and NA are unique moderators of expectancy change during exposure therapy. Third, the deconstruction of within-participant and between-participant effects highlighted how NA and PA differentially moderate expectancy change, leading to unique implications and future directions within the negative versus positive valence systems.

Several limitations constrain the generalizability of this study. First, these findings should be considered preliminary due to the relatively small sample size in each treatment condition. Future research should replicate these findings within a larger sample of anxious individuals, particularly including a wider range of PA (e.g., those experiencing high anhedonia symptoms). Relatedly, the present study was conducted specifically among individuals seeking treatment for social anxiety disorder or panic disorder. Future research should examine associations between affect and expectancy violation across a wider range of anxiety symptoms, comorbid anhedonia symptoms (e.g., major depressive disorder), or symptom severity (e.g., non-clinical samples). Furthermore, in alignment with standard practices in habituation-based exposure, the present study did not measure or evaluate expectancy within the Habituation condition, and exposures were specifically designed using a fear hierarchy (not designed based on expectancy of a feared outcome). This means that fear change is directly built into exposures in the Habituation model (i.e., most participants show high Pre-Fear ratings and a reduction of ≥50% by the Post-Fear ratings), may limit the detection of NA or PA moderation effects during exposures. It is therefore possible that NA and PA are relevant to important exposure processes even when exposures are not explicitly designed around inhibitory retrieval mechanisms. Future research is needed to test this possibility. In addition, although the sample represented a diverse range of racial and ethnic backgrounds, the small sample size and lack of diversity in other areas (e.g., sexual orientation, gender identity, SES) limits the generalizability of these results. Future research is needed to examine how individual difference variables further inform the reported effects. Finally, the present study used data from three time-points for each individual. Future research may gather session-by-session affect ratings to more clearly capture in-the-moment or “state” affect and directly contrast them with trait measures of affect. Likewise, future research may leverage computational modeling approaches within behavioral assays of fear extinction or by analyzing repeated exposure exercises (e.g., during homework), which can further elucidate associations between affect and expectancy violation across numerous trials and timepoints. These approaches may be especially useful for clarifying the extent to which within-person effects are attributable to in-the-moment or “state” levels of affect over and above other factors, such as changes in retrospective reporting biases during the course of exposure therapy.

## 5. Conclusion

In sum, the present study highlights the potential importance of both NA and PA in moderating the learning mechanisms of exposure therapy. Additional research is needed to replicate and extend these findings within a broader population of participants presenting with a wider range of anxiety symptoms.

## Supplementary Material

Supplement_FigS1

AnalysisCode

AnalysisOutput

## Figures and Tables

**Fig. 1.**
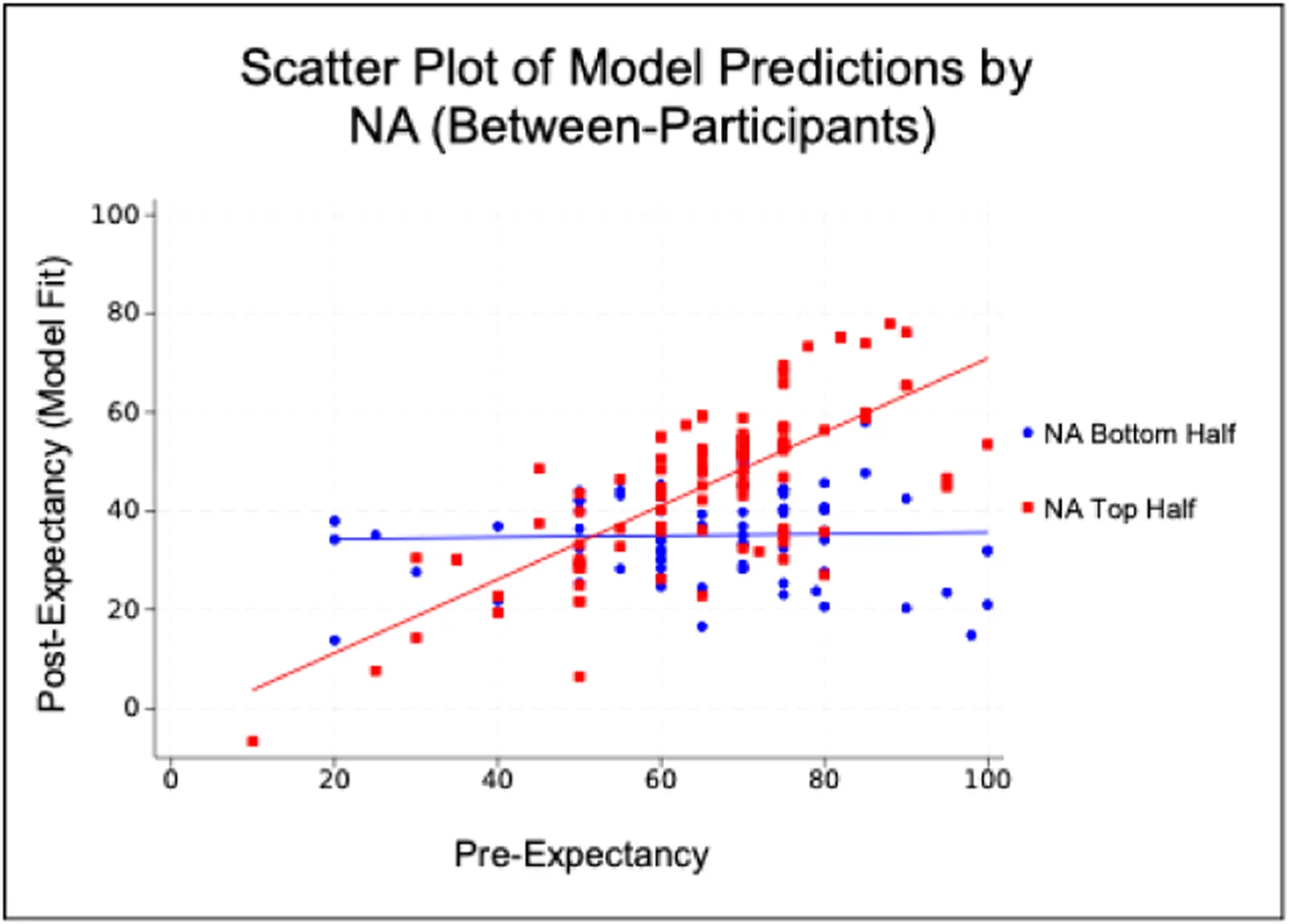
NA moderates expectancy change, such that higher NA (between participants) was associated with less expectancy change during exposures in which there was a high expectancy of an aversive outcome. Although NA is a continuous measure, this figure separates NA values into two groups for ease of interpretability.

**Fig. 2.**
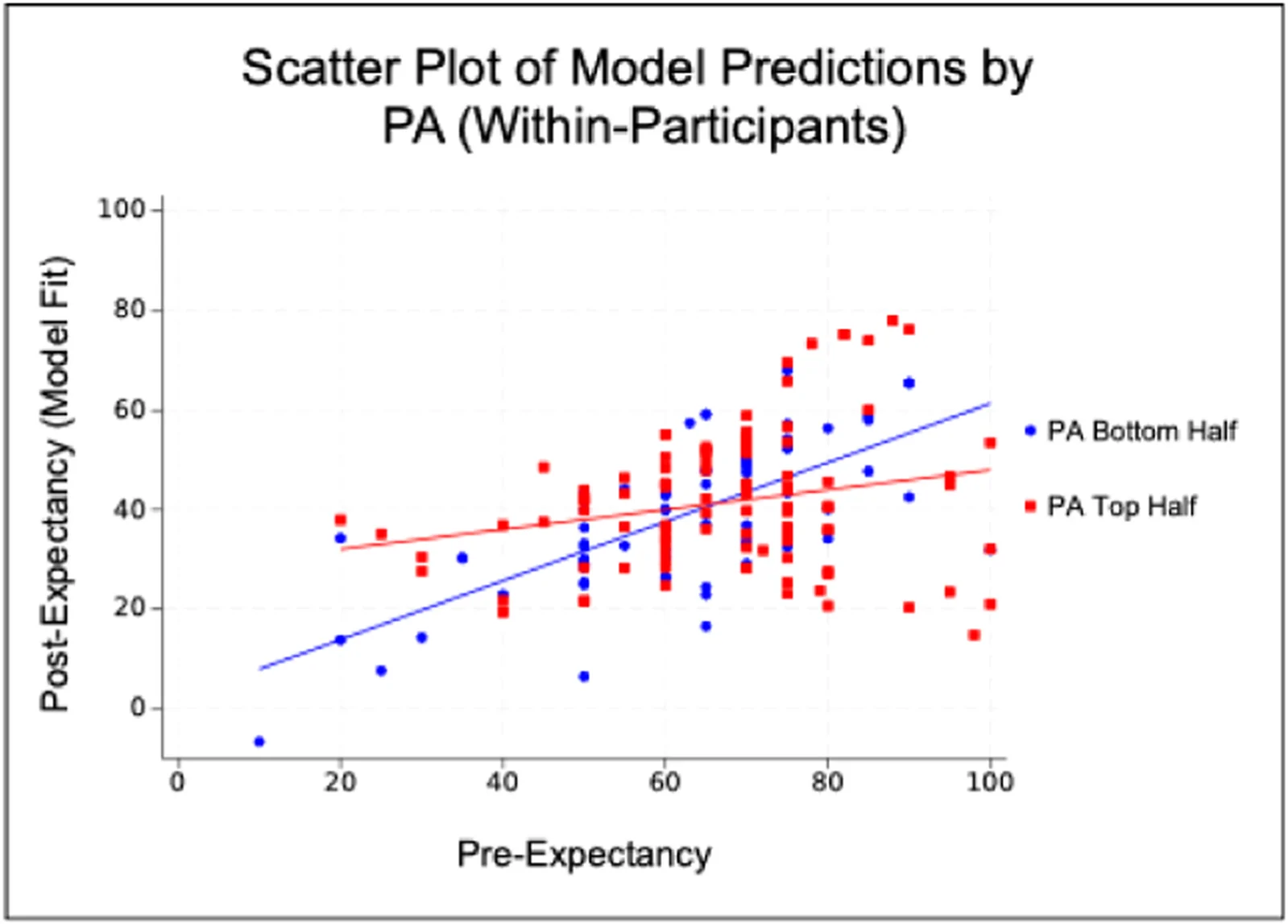
PA moderates expectancy change, such that lower PA (within participants) was associated with less expectancy change during exposures in which there was a high expectancy of an aversive outcome. Although PA is a continuous measure, this figure separates PA values into two groups for ease of interpretability.

**Table 1:** Demographics.

	Inhibitory Retrieval	Habituation	Statistic	*p*-value
N	41	36		
**Sex (N, %)**			X^2^(2)= 1.00	.608
Female	28 (68.29%)	28 (77.78%)		
Male	12 (29.27%)	7 (19.44%)		
Not Reported	1 (2.44%)	1 (2.78%)		
**Age (M, SD)**	26.90 (9.92)	25.67 (11.12)	t = −0.31	.756
**Race (N, %)**			X^2^(8)= 4.27	.832
Alaskan Native/Native American/Indigenous	1 (2.44%)	0 (0%)		
Asian	10 (24.39%)	11 (30.56%)		
Black	0 (0%)	2 (5.56%)		
Latino(a)/Hispanic (Non-White)	5 (12.20%)	5 (13.89%)		
Latino(a)/Hispanic (White)	2 (4.88%)	2 (5.56%)		
Middle Eastern	2 (4.88%)	1 (2.78%)		
White	14 (34.15%)	11 (30.56%)		
Multiracial	5 (12.20%)	3 (8.33%)		
Did not report	2 (4.88%)	1 (2.78%)		
**Current psychotropic medication use (N, %)**			X^2^(2)= 0.02	.990
Yes	14 (34.15%)	12 (33.33%)		
No	25 (60.98%)	22 (61.11%)		
Data not available	2 (4.88%)	2 (5.56%)		

## Data Availability

To enhance transparency, analysis code and corresponding raw outputs are provided in the Supplement. Deidentified data will be made available upon request.

## Footnotes

4 The terms “conditioned” and “conditional” are used interchangeably in the Pavlovian literature when referring to this concept. We prefer the term “conditional” because it emphasizes that the response depends on a learned association between stimuli.

5 Session 2 is used here because exposures were not conducted during session 1.

6 As participants typically completed one exposure per session in the Habituation condition, we did not include the number of exposures completed in each session as a covariate in these analyses.

## Appendix A. Supporting information

Supplementary data associated with this article can be found in the online version at doi:10.1016/j.janxdis.2026.103205.
