# Supplementary material for: Positive and negative affect influence learning during exposure therapy: Secondary analysis from a randomized controlled trial
Source: J Anxiety Disord. Author manuscript; Available in PMC 2026 Nov 9. (PMC13746994; doi:10.1016/j.janxdis.2026.103205)
Supplement: Supplementary file 1 [file NIHMS2209248-supplement-Supplement_FigS1.docx]

**SUPPLEMENT:**

Behavioral Tasks

Several behavioral tasks were collected during the study but were not analyzed for the present report. These included:

- **Generalization of Learning:** This task is designed to measure generalization of acquired fear. It consists of the following types of stimuli: two CS+, one CS-, and eight generalization stimuli (GS’s). This task consisted of three phases: acquisition, extinction and retest.
- **Public Speaking Task:** Participants completed a public speaking task in which they spoke for a short period of time in front of an audience. They were randomly assigned a topic from twelve possible topics and periodically reported their anxiety level throughout the task.
- **Implicit Pavlovian Task:** Participants completed a fifteen-minute computer task in which they were quickly presented a series of words. They were directed to press one of two buttons on a keyboard to indicate if it is a 'word' or a 'non-word', prompting the next word to flash on the screen. The second half of the task repeated the same procedure, but participants were presented two words in quick succession. Participants were instructed to focus their ratings on the second word only, as the first word is used as a distractor.

Measures of NA and PA:

The following table lists the items that were included in the PANAS-P and PANAS-N scales respectively (derived from PANAS-X; Watson & Clark, 1994):

| **PANAS-P** | **PANAS-N** |
| --- | --- |
| Active | Afraid |
| Alert | Alone |
| Attentive | Ashamed |
| Bold | Blue |
| Cheerful | Distressed |
| Concentrating | Downhearted |
| Confident | Frightened |
| Daring | Guilty |
| Delighted | Hostile |
| Determined | Irritable |
| Energetic | Jittery |
| Enthusiastic | Lonely |
| Excited | Nervous |
| Fearless | Sad |
| Happy | Scared |
| Inspired | Shaky |
| Interested | Upset |
| Joyful |  |
| Lively |  |
| Proud |  |
| Strong |  |
| Calm |  |
| Relaxed |  |
| At Ease |  |

This measure of NA was not significantly correlated with PreFear at Session 2, Session 5, or Session 9 (all *p*’s > .29), suggesting that NA in this study is distinct from in-the-moment spikes in NA.

Items Omitted from the PANAS-X

Of the 60 items in the PANAS-X, the following items were not included in the present study: 1) Basic Negative Emotion Scales – Hostility (any items that were not included in in the “General Dimension Scales – Negative Affect” were omitted; *Angry, Scornful, Disgusted, Loathing*), 2) Basic Negative Emotion Scales – Guilt (any items that were not included in the “General Dimension Scales – Negative Affect” were omitted; *Blameworthy, Angry at self, Disgusted with self, Dissatisfied with self*), 3) Other Affective States – Shyness (all items omitted; *Shy, Bashful, Sheepish, Timid*), 4) Other Affective States – Fatigue (all items omitted; *Sleepy, Tired, Sluggish, Drowsy*), 5) Other Affective States – Surprise (all items omitted; *Amazed, Surprised, Astonished*).

Supplemental Figures:

Figure S1: Distribution of NA and PA scores at each timepoint during the RCT.
